# Finding the *K* best synthesis plans

**DOI:** 10.1186/s13321-018-0273-z

**Published:** 2018-04-05

**Authors:** Rolf Fagerberg, Christoph Flamm, Rojin Kianian, Daniel Merkle, Peter F. Stadler

**Affiliations:** 1 Department of Mathematics and Computer Science, University of Southern Denmark, Campusvej 55, 5230 Odense, Denmark; 2 Institute for Theoretical Chemistry, University of Vienna, Währingerstraße 17, 1090 Vienna, Austria; 3 Bioinformatics Group, Department of Computer Science, Interdisciplinary Center for Bioinformatics, Härtelstraße 16-18, 04107 Leipzig, Germany; 4 Max Planck Institute for Mathematics in the Sciences, Inselstraße 22, 04103 Leipzig, Germany; 5 Fraunhofer Institute for Cell Therapy and Immunology, Perlickstraße 1, 04103 Leipzig, Germany; 6 Center for non-coding RNA in Technology and Health, University of Copenhagen, Grønnegårdsvej 3, 1870 Frederiksberg C, Denmark; 7 Santa Fe Institute, 1399 Hyde Park Rd, 87501 Santa Fe, USA

**Keywords:** Synthesis planning, Bond set, Hypergraph, Hyperpath, Algorithm, Convergency, Decalin

## Abstract

In synthesis planning, the goal is to synthesize a target molecule from available starting materials, possibly optimizing costs such as price or environmental impact of the process. Current algorithmic approaches to synthesis planning are usually based on selecting a bond set and finding a single good plan among those induced by it. We demonstrate that synthesis planning can be phrased as a combinatorial optimization problem on hypergraphs by modeling individual synthesis plans as directed hyperpaths embedded in a hypergraph of reactions (HoR) representing the chemistry of interest. As a consequence, a polynomial time algorithm to find the *K* shortest hyperpaths can be used to compute the *K* best synthesis plans for a given target molecule. Having *K* good plans to choose from has many benefits: it makes the synthesis planning process much more robust when in later stages adding further chemical detail, it allows one to combine several notions of cost, and it provides a way to deal with imprecise yield estimates. A bond set gives rise to a HoR in a natural way. However, our modeling is not restricted to bond set based approaches—any set of known reactions and starting materials can be used to define a HoR. We also discuss classical quality measures for synthesis plans, such as overall yield and convergency, and demonstrate that convergency has a built-in inconsistency which could render its use in synthesis planning questionable. Decalin is used as an illustrative example of the use and implications of our results.
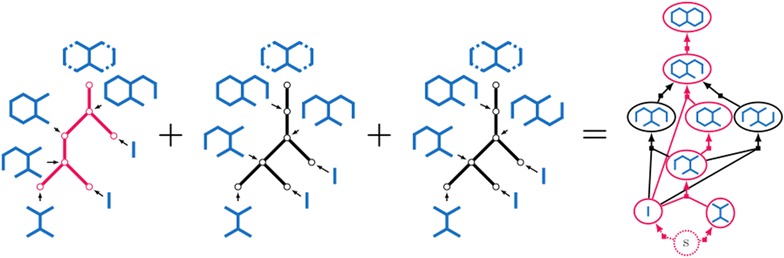

## Introduction

Synthesis planning is a core problem in chemistry, first treated as computational problem by Corey [1] in the late sixties. The objective is to find a way to synthesize a given target molecule from available starting materials, possibly optimizing costs such as amount of materials, price, or environmental impact of the process.

Synthesis planning is still regarded as somewhat of an art form, although attempts have been made over several decades at applying formal approaches and computational methods [1–15]. Such attempts have focused on models of synthesis plans, quality measures for ranking such plans, and algorithms for finding the best plan among several possible plans.

An early contribution towards an automated approach for synthesis planning is retrosynthetic analysis. It was introduced by Corey [1, 12, 13] in 1969 as part of a formalization of the rules of synthesis used in the development of the computer program LHASA (logic and heuristics applied to synthetic analysis). Retrosynthetic analysis is a top-down approach to synthesis planning, which governs the selection of the chemical bonds to be involved in the synthesis by using heuristics formulated to mimic a chemist’s reasoning. The basic idea is simple: Starting with the target chemical structure, split the molecule by removing a bond indicated by the heuristic. Then recursively continue on the generated smaller molecules until sufficiently simple or commercially available starting materials have been found. As an alternative to using heuristics trying to mimic the choices of chemists, Bertz [6, 10] suggested choosing bonds by minimizing the graph theoretical molecular complexity of the resulting precursor molecules. Other approaches include choosing bonds based on the existence of substructures of the target molecule that are isomorphic to easily accessible or available molecules found in libraries [16–19]. An overview of programs for synthesis planning based on retrosynthesis can be found in [3, 14].

A major drawback of retrosynthesis, however, is that it is a greedy approach. In the attempt to make good choices during the retrosynthetic top-down recursion, it leaves out synthesis plans with costly last steps but much better first steps. Consequently, plans found using a retrosynthetic approach are not necessarily optimal plans according to quality measures for synthesis plans.

## Contribution

The starting point of this paper is the observation that computing a *single* good plan does not always suffice: Models of synthesis plans and definitions of quality measures necessarily leave out many real-world details, and the best plan according to a given choice of model and quality measure may turn out to be infeasible when later adding further chemical details to the plan. A more robust strategy would be to instead find a *number* of good plans from which the practitioner can choose based on additional chemical knowledge and wet-lab feasibility. We believe that such a strategy can significantly increase the practical value of formal synthesis planning. Generating and evaluating all possible plans is a natural approach, but this is highly inefficient due to the combinatorial explosion in the number of plans. In this paper, we make the strategy feasible by providing an efficient method for finding the *K* best synthesis plans, for any number *K*.

Our approach is based on representing the set of chemical reactions under interest as a directed hypergraph (a known method in chemistry, see e.g. [9]). We demonstrate that synthesis plans correspond exactly to the concept of hyperpaths^1^ in such hypergraphs. This in turn allows us to exploit an existing polynomial time algorithm [20] for finding the *K* shortest hyperpaths in a hypergraph—to our knowledge the first use of this algorithm in a chemical context. The result is a big improvement in the computational complexity of ranked enumeration of synthesis plans, which is our core contribution.

Besides adding robustness to synthesis planning, the strategy also enables an easy way to find plans that are optimized according to more than one quality measure: compute a set of the best plans for each measure and output their intersection. Similarly, one can use intersections of sets of good plans for several values of yield estimates, in order to obtain plans stable against variance in the actual yields obtained. Another feature of our approach is that it is not restricted to using a so-called bond set (many existing methods in formal synthesis planning are based on bond sets), but it has larger flexibility in terms of modeling the set of reactions available.

Along the way, we also demonstrate that one of the known classical quality measures surprisingly has a built-in inconsistency which could render its use in synthesis planning questionable.

The rest of this paper is structured as follows: In the “Previous work” section, we list the existing research closest to our contribution. In the “Synthesis planning basics” section, we outline relevant synthesis planning concepts. In the “Results” section, we point out problems that can arise when using unary-binary trees for modeling synthesis plans, and demonstrate how to solve these problems by the use of directed acyclic graphs and hypergraphs. We define a structure called a hypergraph of reactions, which we prove contains all synthesis plans as hyperpaths (Theorem 1). We then show how this allows us to find the *K* best synthesis plans in polynomial time (Theorem 2). We also discuss quality measures. In the “Discussion” section, we put our approach in a practical context using the molecule decalin as an example, before ending with "Conclusions" section.

## Previous work

The previous line of work closest to the work in this paper is by Hendrickson [8] and by Smith [9]. They both focus on graph based descriptions of synthesis plans, and on formal quality measures of these plans. Hendrickson [8] models synthesis plans as binary trees, and defines quality measures based on convergency [21], which essentially is how balanced the tree is. The rationale for this quality measure is that the more balanced the tree for a plan is, the fewer reactions the average starting material will take part in, either directly or as part of larger molecules in later reactions. All reactions incur some loss, and the strategy aims at reducing this loss. Smith [9] models synthesis plans as hypergraphs, and defines quality measures more explicitly based on the actual loss incurred by each reaction. In both papers, the focus is on finding a single best plan according to the quality measure in question. Smith explicitly gives an algorithm based on dynamic programming for finding this in his setting.

In terms of programs for synthesis planning, Hendrickson’s line of work has led to a program SynGen (synthetic generator) [15]. This program retrosynthetically expands all possible ways to synthesize the carbon skeleton of the target molecule, offering different ways to assist selection of plans after they have been computed. As the program enumerates *all* plans, it is computationally costly even for synthesis plans of depth three [15].

Our work in this paper can be said to extend the methods of Smith and Hendrickson. In combination with an algorithm from [20], we are able to find the *K* best synthesis plans for a target without having to compute all plans, resulting in a much better computational efficiency.

A concept somewhat related to synthesis planning is finding pathways in metabolic networks, see e.g. [22] for a recent review. The work most closely related to ours is [23], which models metabolic pathways as a type of hyperpaths and gives a method for enumerating pathways between a set of source compounds and a target compound in the network. However, their algorithm enumerates all pathways, not the *K* best according to a quality measure. Other papers reviewed in [22] do consider finding pathways in ranked order, but the modeling there is as simple paths in standard graphs.

## Synthesis planning basics

A synthesis plan describes a way to synthesize a given target molecule from available starting materials by a set of chemical reactions. There are two main types of reactions [8]: *construction* reactions, which create new carbon–carbon bonds in the target’s skeleton, and *functionalization* reactions, which alter the functional groups attached to the skeleton but do not alter the skeleton itself. Synthesis planning often proceeds in two phases, the first of which is identification of a set of construction reactions, and the second is consideration of functionalization reactions. The rationale for using such a two phase procedure (from fewer to more chemical features) during the search for an optimal synthesis plan is that the full chemical design space is too vast to explore efficiently. Usually, the first phase is considered to be the core of the planning—to quote Hoffmann [7, p. 5], *“The points stressed earlier should be highlighted once more: Construction of the skeleton of the target structure is the prime task in synthesis planning, not the placement of functionalities or stereogenic centers”*. Following most of the work on formal methods for synthesis planning, we therefore focus on the first phase and model construction reactions only.

Construction reactions come in two variants: *affixations* which add target bonds by uniting two separate precursor molecules, and *cyclizations* which add target bonds by closing a ring in a single molecule. Construction reactions are often said to *fix* a bond in the target, and the set of bonds fixed in the plan is denoted the *bond set*.

**Fig. 1:**
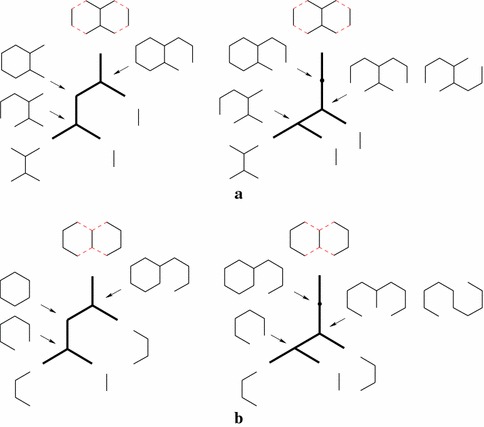
Example synthesis plans for decalin for two different bond sets of size four. The bonds in each bond set are red and dashed. Leaf vertices are labeled with starting materials, internal vertices are labeled with intermediate molecules. As a shorthand, each of the rightmost trees has an internal vertex labeled with two molecules and represents two different plans

The usual way to depict such synthesis plans is using unary-binary trees, where affixations give rise to binary vertices and cyclizations to unary vertices. Leaves represent starting materials, internal vertices represent intermediate molecules, and the root represents the target molecule. Some example plans for the target molecule decahydronaphtalene (IUPAC name Bicyclo(4.4.0)-decane) are depicted in Fig. 1. This compound, with the trade name decalin^®^, is a bicyclic organic molecule with ten carbon atoms. It is an important industrial solvent for resins, waxes, and oils, and it serves as a component in jet fuels. The rightmost synthesis plans in Fig. 1 start with two affixations followed by two cyclizations. The leftmost plans alternate between an affixation and a cyclization. The bonds in the bond set are red and dashed.

Obviously, there can be several plans for the same target molecule. As an example, one can consider the bond set as a summary of the synthesis plan [8], actually representing a larger set of alternative synthesis plans arising from considering different orders of fixing the bonds. These plans may differ substantially in their yield, lab resource consumption, and environmental side effects. Thus, for a given set of synthesis plans there is a need to find the best. To this end, several quality measures for ranking synthesis plans have been proposed. Two classical ones are the *external path length* (${{\mathrm{EPL}}}$) and *total weight of starting materials* (${{\mathrm{TW}}}$).

The measure ${{\mathrm{EPL}}}$ was introduced by Hendrickson [8], and is defined as the sum of the numbers of reactions from each starting material to the target. When modeling synthesis plans as unary-binary trees, this is the sum of the lengths of all root-to-leaf paths, which is also known as the external path length of the tree. This measure optimizes the convergency [21] of the plans: fully convergent plans (balanced trees) minimizes ${{\mathrm{EPL}}}$, whereas linear plans maximizes it. The measure aims at reducing overall loss of material during the synthesis by reducing the number of reactions in which the average starting material takes part. However, we demonstrate later in the paper that this classic quality measure has intrinsic inconsistencies which could make its use questionable.

The measure ${{\mathrm{TW}}}$ is defined as the total weight of starting materials in grams needed to produce one gram of the target molecule, and hence is a more direct description of the overall loss of material. This measure was described by Hendrickson [8] and later by Smith [9]. The two authors differ in the way the value is calculated. In “Appendix 4: Total weight of starting materials” we demonstrate that Smith’s definition is in fact a generalization of that of Hendrickson, hence we here focus on the definition by Smith.

In the unary-binary tree representation of a synthesis plan, an edge *e* in the tree connects an input molecule *v* of a reaction with its output molecule *u*. Smith assumes knowledge of the loss in each individual reaction, and expresses this by values $r_e$ on all edges *e*, where $r_e$ is the amount in grammes of molecule *v* needed to create one gram of molecule *u*. Let $P_{i}$ be the path from the root to leaf *i*. The total weight of starting material *i* needed to produce one gram of target is then equal to the product of the $r_e$ values along $P_i$. Thus, the total weight of starting materials needed to produce one gram of target is the sum of these values over all paths. Hence,

1 $$\begin{aligned} {{\mathrm{TW}}}= \sum _{i} \prod _{e\in P_{i}} r_e \end{aligned}$$

As Smith notes, by adding virtual unary reactions below all leaves with $r_e$ values signifying price per gram, ${{\mathrm{TW}}}$ can easily express price rather than weight. In ${{\mathrm{TW}}}$, the cost of a reaction is measured per gram of output molecule produced, i.e., upstart costs of reactions are not accounted for. Hence, the quality measure is measuring the asymptotic cost when large amounts of the target molecule are to be produced.

## Results

### Representations of synthesis plans

In a synthesis plan, an intermediate molecule may be used as input in several reactions. Our starting point is the observation that in such cases, this intermediate molecule clearly should only be synthesized in one way: given two different ways to synthesize a given intermediate molecule, one will have the smallest asymptotic cost, and even if the two have equal costs, using both induces extra overhead.

**Fig. 2:**
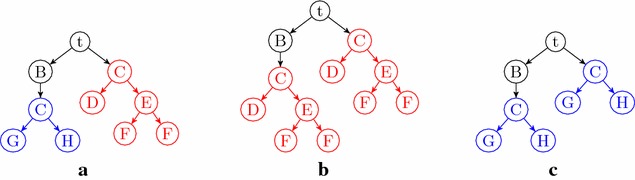
Unary-binary trees representing synthesis plans. Vertices with identical labels represent identical molecules. **a** A tree with two different subtrees for vertices with label *C*. Substituting one subtree for the other results in the tree **b** or the tree **c**

This means that if an intermediate molecule appears more than once as a vertex in a unary-binary tree representation of a synthesis plan, its subtrees should be identical. To illustrate, in the tree in Fig. 2a, molecule *C* is synthesized in two different ways. However, either the tree Fig. 2b or the tree Fig. 2c must be cheaper, depending on the costs of the two ways of synthesizing *C*. In short, while synthesis plans can be represented by unary-binary trees with nodes labeled by molecules, not all such unary-binary trees are realistic synthesis plans.

**Fig. 3:**
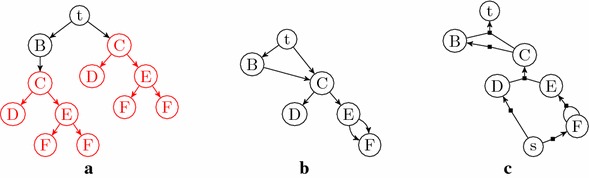
Three different ways to model synthesis plans. In the unary-binary representation, the subtrees rooted in *C* are identical and are thus merged in the DAG and hyperpath representations. **a** Traditional notation. **b** DAG. **c** Hyperpath

As a consequence, we believe synthesis plans are better described as directed acyclic graphs (DAGs) in which each vertex has a unique vertex label, each vertex has out-degree zero, one or two (depending on whether it represents a starting material, a product of a cyclization, or a product of an affixation, respectively), there is exactly one vertex *t* with in-degree zero (representing the target molecule), and there is a path from *t* to any other vertex in the DAG. Such a DAG can be obtained from the unary-binary tree structure by merging vertices with the same label. Vice versa, a tree structure can be obtained from the DAG by a depth-first search from the root of the DAG, in a version which allows revisits to vertices. In Fig. 3b, the DAG arising from the unary-binary tree in Fig. 3a is shown.

In the DAG (as well as in unary-binary trees), labels of vertices are molecules. For this labeling to be chemically meaningful, the labels cannot be arbitrary, but should reflect the reactions involved. We now formalize this, using graph models of molecules as labels.

If edge (*v*, *u*) is in the DAG, we denote vertex *u* a child of *v*. A vertex with out-degree zero we denote a leaf. A *molecule* is an undirected, connected, labeled graph, where labels are atom types. A *building block* of a DAG is a non-leaf vertex *v* together with every child *u* of *v* and its corresponding edge (*v*, *u*). In a DAG with nodes labeled by molecules, a building block is a *reaction* if it satisfies the following: (1) *v* has one or two children. (2) If *v* has one child *u*, the label of *v* is obtained from the label of *u* by adding exactly one edge. This is a cyclization. (3) If *v* has two children $u_1$ and $u_2$, then the label of *v* is obtained from the labels of $u_1$ and $u_2$ by adding exactly one edge connecting these labels. This is an affixation. A *starting material* is a molecule that can be acquired without the need to plan how to synthesize it.

#### **Definition 1**

A *synthesis plan* for *t* is a labeled DAG with the following properties:

1. Vertex labels are molecules, and each label is unique.
2. Each building block is a reaction.
3. There is exactly one vertex with in-degree zero, namely *t*.
4. The label of each leaf is a starting material.

Our next observation is that such DAG models of synthesis plans can be represented as directed *hypergraphs*. A hypergraph differs from a standard graph in that edges connect sets of vertices rather than single vertices. More precisely, a *hyperarc* is an ordered pair of vertex (multi)sets. In chemistry, a reaction is a multiset of reactants and a multiset of products, and can therefore be seen as a hyperarc. As we only model construction reactions, all heads are singletons in this paper. In Fig. 3c, the reactions of Fig. 3b are depicted as hyperarcs.

When we say that a hyperarc represents a chemical reaction, we mean that its vertices are labeled with molecules, and that the label of the head is obtained from the labels of the tail under the reaction in question, analogously to the definition of a reaction in a DAG given above.

Hypergraphs are well suited for modeling chemistry, because they make the relationship between all molecules involved in a reaction explicit. This is in contrast to a DAG or a tree, where the two reactants in a reaction are only indirectly related via their common product.

In a hypergraph representation of the synthesis plan, adding a dummy source vertex *s*, together with a directed hyperarc $(\{s\}, \{i\})$ to each starting material *i* of the synthesis plan (as shown in Fig. 3c), gives a hypergraph which is in fact a *hyperpath*. This allows us to think of a synthesis plan as a form of path, hence to think of optimal synthesis plans as optimal paths. Given a larger hypergraph modeling the reactions in a part of chemistry under consideration, the search for optimal synthesis plans within this chemistry then becomes a search for optimal paths in the hypergraph.

Below, we recap basic hypergraph terminology, and then show how to use hypergraphs for finding synthesis plans.

### Hypergraphs

A directed hypergraph is a set *V* of vertices and a set *E* of *hyperarcs*, where each hyperarc $e=(T(e), H(e))$ is an ordered pair of non-empty multisets of vertices. The set *T*(*e*) is denoted the *tail* of the hyperarc and *H*(*e*)) the *head*. If $|H(e)| = 1$, the hyperarc is denoted a *B-hyperarc*, and the notation for the single head vertex is *h*(*e*). A hypergraph with only B-hyperarcs is denoted a *B-hypergraph*. In this paper, we only consider B-hypergraphs. The *size* of a B-hypergraph is given by ${{\mathrm{size}}}(H)=\sum _{e\in E}(|T(e)| + 1)$. A hypergraph $H'=(V',E')$ is a *subhypergraph* of $H=(V,E)$ if $V'\subseteq V$ and $E'\subseteq E$.

A (plain) *path* $P_{st}$ from *s* to *t* in a B-hypergraph is a sequence $P_{st}=\langle s, e_1, v_1, e_2, v_2, \dots , v_{q-1}, e_q,t \rangle$ of vertices and B-hyperarcs such that $s \in T(e_1)$, $t=h(e_q)$ and $v_i=h(e_i) \in T(e_{i+1})$ for $i=1..q-1$. Its length $|P_{st}|$ is the number *q* of hyperarcs. If $t\in T(e_1)$, then $P_{st}$ is a *cycle*. A hypergraph is *acyclic* if it does not contain any cycles.

The above concept of paths is only used for defining cycles. The proper generalization of directed paths to hypergraphs is that of *hyperpaths*. There are different ways of defining this—we use a variation based on [24]. For a general overview see [25]. Examples of what constitutes a hyperpath and what does not are given in “Appendix 1: Hyperpaths”.

#### **Definition 2**

A *hyperpath* $\pi _{st}=(V_\pi , E_\pi )$ from a source vertex *s* to a target vertex *t* in a B-hypergraph *H* is a subhypergraph of *H* with the following properties: If $t=s$, then $V_\pi = \{s\}$ and $E_\pi = \emptyset$. Otherwise, $E_\pi$ can be ordered in a sequence $\langle e_1, e_2, \ldots , e_q \rangle$ such that

1. $$T(e_i)\subseteq \{s\} \cup \{h(e_1), h(e_2), \ldots , h(e_{i-1})\}$$ for all *i*
2. $$t=h(e_q)$$
3. Every $v \in V_\pi {\setminus } \{ t \}$ has at least one outgoing hyperarc in $E_\pi$, and *t* has zero.
4. Every $v \in V_\pi {\setminus } \{s\}$ has exactly one ingoing hyperarc in $E_\pi$, and *s* has zero.

Note that Definition 2(4) gives a 1–1 correspondence between $E_\pi$ and $V_\pi {\setminus } \{s\}$, hence we can define unique indices for the vertices in $V_\pi {\setminus } \{s\}$ by $v_i = h(e_i)$. We let $v_0$ be *s*. The hyperarc $e_i$ is called the *predecessor* hyperarc of $v_i$ and the corresponding map $p: V_\pi {\setminus } \{s\} \mapsto E_\pi$ is called the predecessor function of $\pi _{st}$ [20]. We use the notation $\pi _{st} = \langle p(v_1), p(v_2),\ldots ,p(v_{q-1}), p(t) \rangle$ for hyperpaths from now on. If a subhypergraph of a hyperpath $\pi _{st}$ is a hyperpath itself, it is called a *subhyperpath* of $\pi _{st}$. We will later need the following fact.

#### **Lemma 1**

*Let *$\pi _{st} = \langle p(v_1), p(v_2),\ldots ,p(v_{q-1}), p(t) \rangle$* be a hyperpath from* *s* to *t*.* Then for any *$v_i$,* there is a unique subhyperpath* $\pi _{sv_i}$ of $\pi _{st}$* from* *s* to $v_i$.

#### *Proof*

By Definition 2(2) and (4), any subhyperpath from *s* to $v_i$ must contain the set $\pi _{sv_i}$ of hyperarcs returned by the procedure Backtrack listed in Algorithm 1. It is easy to verify that $\pi _{sv_i}$ fulfills the requirements Definition 2(1)–(4). For uniqueness, let $\pi '_{sv_i}$ be a hyperpath from *s* to $v_i$, let $E'$ be the hyperarcs of $\pi '_{sv_i}$ not in $\pi _{sv_i}$, and let $e_j$ be the hyperarc of $E'$ with highest index *j*. By Definition 2(3), $v_j$ must have an outgoing hyperarc *e*. By Definition 2(1) and the maximality of *j*, we cannot have $e \in E'$, and by the marking strategy of the algorithm, it cannot be a hyperarc of $\pi _{sv_i}$. Hence, $E'$ is empty and $\pi _{sv_i} = \pi '_{sv_i}$. $\square$

**Figure:**
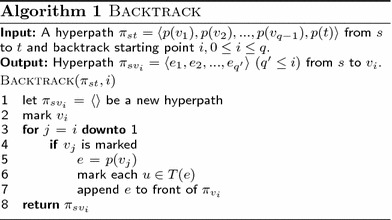


### Finding synthesis plans via hypergraphs

The overall goal of this paper is to find synthesis plans within a given chemistry. We assume the chemistry is described by a (possibly large) set of construction reactions, i.e., affixations and cyclizations. Above, we described how to model synthesis plans as hypergraphs. In this section, we show how to view the set of reactions of the given chemistry as a single, large hypergraph, and how this in turn will allow us to find synthesis plans within the chemistry simply by looking for hyperpaths in this hypergraph.

Let *R* be a set of reactions, and let *S* be a set of starting materials. We define the hypergraph, called *the Hypergraph of Reactions* (${{\mathrm{HoR}}}$) induced by *R* and *S*, as follows.

#### **Definition 3**

Let $E_R$ be the representation of *R* as a set of hyperarcs. Let $V_R$ be the set of vertices appearing in the heads and tails of the hyperarcs in $E_R$. Vertices with the same label (i.e., representing the same molecule) are considered identical. Let $V_S$ be the set of vertices with labels in *S*. Then, the *hypergraph of reactions* (${{\mathrm{HoR}}}$) is the hypergraph

$$H=(V_S \cup V_R\cup \{s\}, E_R\cup E_s),$$

where *s* is a dummy vertex and $E_s = \{ (\{s\},\{v\}) \mid v \in V_S \}$ is a set of dummy hyperarcs.

**Fig. 4:**
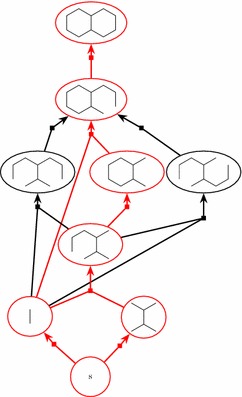
An example ${{\mathrm{HoR}}}$ based on the combined sets of reactions and starting materials from the three synthesis plans for decalin in Fig. 1a. The hyperpath corresponding to the leftmost tree in Fig. 1a is highlighted in red

An example ${{\mathrm{HoR}}}$ with *R* and *S* being the combined sets of reactions and starting materials from the three synthesis plans for decalin in Fig. 1a is depicted in Fig. 4.

The crux of our paper is captured by the following theorem.

#### **Theorem 1**

*Let* $H=(V,E)$ *be a HoR induced by reactions R and starting materials S. Let v be a vertex from* $V_R$ *and let t be its label. Then there is a 1-1 correspondence between (a) the synthesis plans for t with reactions from R and starting materials from S and (b) the hyperpaths in H from s to v.*

#### *Proof*

Let $\sigma$ be a synthesis plan from (a). To map it to a hyperpath in (b), do as follows. Add a dummy vertex *s* and a dummy edge from each leaf of $\sigma$–*s*. This result is still a DAG, hence admits a topological ordering of its vertices, i.e., a linear order on its vertices such that all edges point in the same direction. Convert each reaction and dummy edge of the DAG into a hyperarc in the natural way, cf. Fig. 3, reversing the direction. Let $E_{\pi }$ be the resulting hyperarcs, and let $V_{\pi }$ be the vertices of the DAG. Then $\pi = (V_{\pi }, E_{\pi })$ is a hyperpath: Definition 2(4) follows because a building block of a DAG vertex contains all the outgoing DAG edges of the vertex. Due to Definition 2(4), the topological ordering of the DAG vertices induces an ordering of $E_{\pi }$ fulfilling Definition 2(1). Definition 1(iii) induces Definition 2(3) and, combined with the topological ordering, also Definition 2(2).

Conversely, let $\pi$ be a hyperpath from (b). To map it to a synthesis plan in (a), do as follows: From $\pi$ remove *s* and its outgoing dummy hyperarcs, and convert every hyperarc to a building block in the natural way, reversing the direction. The result is a synthesis plan: Definition 1(i) and (ii) follow by what it means for a hyperarc to represent a reaction and by the uniqueness of vertex labels in Definition 3. Definition 1(i) follows from Definition 2(3). Definition 1(iv) follows from Definition 2(4) and the fact that only dummy hyperarcs have been removed, each of which by definition points to a starting material.

Clearly, the two mappings are each other’s inverses.$\square$

As a consequence of Theorem 1, algorithms computing hyperpaths in hypergraphs are also algorithms computing synthesis plans. In particular, we claim that an algorithm by Nielsen et al. [20] for finding the *K* shortest hyperpaths in a B-hypergraph can be used to find the *K* best synthesis plans for a target molecules *t*, given a set of reactions and a set of starting materials. We now verify the details of this claim.

The algorithm by Nielsen et al. is based on Yens classic algorithm [26] for shortest paths in directed (standard) graphs. The overall idea is to find the single shortest hyperpath and then recursively consider all ways in which a hyperpath can deviate from the shortest hyperpaths found so far, using single shortest hyperpath computations as a subroutine. For an acyclic B-hypergraph $H=(V,E)$, the single shortest hyperpath can be computed using dynamic programming [9] in $O(|V|+{{\mathrm{size}}}(H))$ time, leading to a runtime for the algorithm by Nielsen et al. of $O(K|V|(|V|+{{\mathrm{size}}}(H)))$ on such hypergraphs. The requirement of acyclicity can be lifted (at the expense of a slight increase in runtime) by using different algorithms for finding single shortest hyperpaths [27], whereas the algorithm by Nielsen et al. assumes the hypergraph to be a B-hypergraph.

Any ${{\mathrm{HoR}}}$ *H* is acyclic and satisfies ${{\mathrm{size}}}(H)\le 3|E|$. The former is because every reaction has a strict increase from reactants to product in the number of edges in the molecule labels, and the latter is because every hyperarc *e* in a ${{\mathrm{HoR}}}$ is a B-hyperarc with $|T(e)| \le 2$.

Finally, the algorithm by Nielsen et al. requires the lengths of hyperpaths to be given by what is called an additive weight function. We will later demonstrate that the very generic quality measure ${{\mathrm{TW}}}$ for synthesis plans can be expressed in this form.

These properties combined with Theorem 1 give the following result.

#### **Theorem 2**

*Given a HoR* $H = (V,E)$ *and a target* $t \in V$*, the K best synthesis plans for t in H, ranked according to the measure TW, can be found in time* $O(K|V|(|V|+|E|))$.

A detailed exposition of the algorithm by Nielsen et al. is given in “Appendix 2: The *K* best plans algorithm”.

The sets *R* and *S* of reactions and starting materials in the ${{\mathrm{HoR}}}$ can arise from many sources. As in Fig. 4, a set of known synthesis plans for a target *t* can be combined to a ${{\mathrm{HoR}}}$ which could then contain further, unknown plans. Another approach could be to generate the reactions of the ${{\mathrm{HoR}}}$ by recursively breaking the bonds of the target *t* in all possible ways. This could be all bonds of *t* (possibly stopping the recursion when a specified minimum size of intermediate molecules is met), or it could be a subset of bonds (i.e., a bond set) selected by methods from the classic retrosynthetic approach [1]. In “Appendix 3: Bond set based HoR construction”, we provide the algorithmic details of efficiently breaking a bond set in all possible ways. More generally, any database of reactions and starting materials describing a chemistry under consideration can be used as *R* and *S*. These databases can be based on published literature and patents, such as Reaxys [28] and SciFinder [29]. Recent developments for retrosynthetic reaction prediction [30–32] even allow for the inference of unknown reactions. As these automated prediction methods have already proven to be highly accurate, finding the *K* shortest hyperpaths also has potential for discovering novel synthesis plans employing such predicted reactions.

### Quality measures

The algorithm of Nielsen et al. requires the lengths of hyperpaths to be given by an additive weight function. In this section, we investigate whether existing quality measures for synthesis plans can be expressed in this form, and we show that the very generic measure ${{\mathrm{TW}}}$ indeed can. On the other hand, this turns out to not be the case for the classic measure ${{\mathrm{EPL}}}$. The reasons are inconsistencies that we demonstrate are inherent in the measure. This is a bit surprising in light of its use in previous work, but the implication seems to be that the ${{\mathrm{EPL}}}$ measure should be used with caution.

An *additive weight function* *W* assigns weights to hyperpaths in an inductive manner. For our purposes, we only need a special case, often denoted a *value* function (for a more general definition of additive weight functions, see [27]): For each hyperarc *e* and each vertex *v* in its tail *T*(*e*), let $a_{v,e}$ be a non-negative real number. Then for a hyperpath $\pi _{st}$ from *s* to *t*, the weight $W(\pi _{st})$ is one if $t = s$, and is otherwise given recursively by

2 $$\begin{aligned} W(\pi _{st}) = \sum \limits _{v\in T(p(t))} a_{v,p(t)} W(\pi _{sv}), \end{aligned}$$

where the $\pi _{sv}$’s are the subhyperpaths from *s* to the vertices *v* in the tail of last hyperarc *p*(*t*) of $\pi _{st}$. These subhyperpaths exist and are unique by Lemma 1. They also contain strictly fewer hyperarcs, so the recursion stops eventually. Hence, *W* is well-defined.

**Fig. 5:**
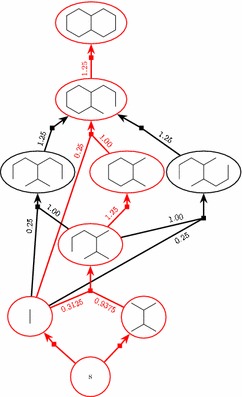
The HoR from Fig. 4 decorated with example retro yields

*Total weight of starting materials* We defined the quality measure total weight of starting materials (${{\mathrm{TW}}}$) in “Synthesis planning basics” section, expressed in unary-binary trees. Recall that ${{\mathrm{TW}}}$ expresses how much starting material is needed to produce one gram of target molecule, taking yields of reactions into account. In the hyperpath setting, the definition becomes the following. For a reaction *e* with a reactant *v*, the *retro yield* $r_{v,e}$ is the amount of *v* in grams needed in reaction *e* to create one gram of the product *h*(*e*). Thus, $r_{v,e}\ge 0$, and by mass conservation $\sum _{v}{r_{v,e}}\ge 1$ for any reaction *e*. Figure 5 shows the HoR from Fig. 4 decorated with example retro yields.

Each (plain) *st*-path $P_{st}=\langle s,e_1,v_1,e_2,v_2,\ldots ,e_{|P_{st}|}, t\rangle$ contained in the hyperpath of the synthesis plan induces a use of starting material $v_1$ given by the product of the retro yields along the path. The product along a path is $\prod _{i=1}^{|P_{st}|}{r_{v_{i-1},e_{i}}}$, where we for each hyperarc *e* from the dummy vertex *s* to a starting material define $r_{s,e} = 1$. Thus, the total weight of starting materials needed for a synthesis plan represented by a hyperpath $\pi _{st}$ is

3 $$\begin{aligned} {{\mathrm{TW}}}(\pi _{st}) = \sum _{P_{st} \,\text {in}\, \pi _{st}}{\prod _{i=1}^{|P_{st}|}{r_{v_{i-1},e_{i}}}} \,. \end{aligned}$$

This can be rephrased inductively as follows

4 $$\begin{aligned} {{\mathrm{TW}}}(\pi _{st}) = \left\{ \begin{array}{ll} 1 &{}\quad \text{ if } \;t=s \\ \sum \limits _{v\in T(p(t))}{r_{v,p(t)}{{\mathrm{TW}}}(\pi _{sv})} &{}\quad \text{ otherwise } \end{array} \right. \end{aligned}$$

This is most easily seen in the traditional tree notation for synthesis plans, cf. Fig. 3. Thus, ${{\mathrm{TW}}}$ can indeed be expressed as an additive weight function, cf. Eq. (2). The measure ${{\mathrm{TW}}}$ is actually very generic in nature [9]. For example, it can easily be adjusted to calculate the total *price* of the starting materials if a price per gram $p_v$ is known for each starting material *v*, simply by setting $r_{s,e} = p_v$ for the hyperarc *e* from *s* to *v*. It can also incorporate non-chemical expenses of reactions, such as cost of energy usage or cost of disposal of waste products, simply by adding *s* to the tail of any hyperarc *e* representing a reaction and setting $r_{s,e}$ to the non-chemical expense per gram product produced in *e*. Even more generally, we note that any measure which can be described as an additive weight function [27] is compatible with our method.

**Fig. 6:**
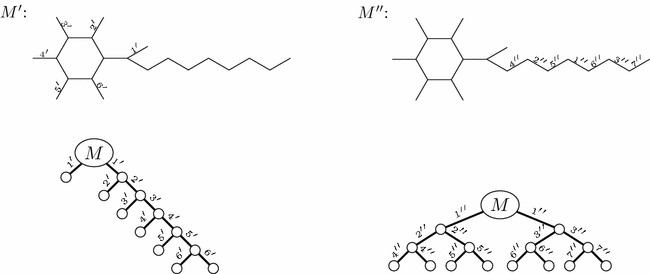
The top row shows a hypothetical molecule *M* with two different bond sets $M'$ and $M''$. The bonds in the bond sets are the edges labeled by numbers. $M'$ admits only linear synthesis plans, whereas $M''$ admits many, including a fully convergent one. The bottom row shows a linear plan based on $M'$ and a fully convergent plan based on $M''$

**Fig. 7:**
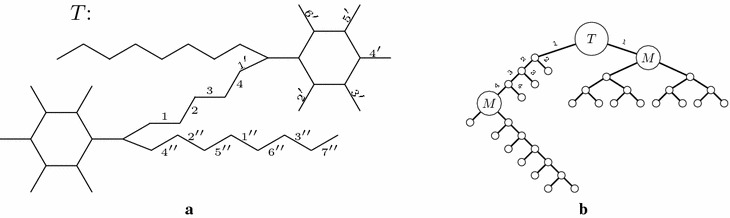
A molecule *T* is shown in **a** with a bond set consisting of the labeled edges. Note that the molecule *M* appears twice as a substructure in *T*, and that the bond sets $M'$ and $M''$ are subsets of the bond set of *T*. A corresponding synthesis plan for *T* is shown in **b**. The ${{\mathrm{EPL}}}$ for this plan is 96. **a** Molecule *T*. **b** A synthesis plan for *T*

**Fig. 8:**
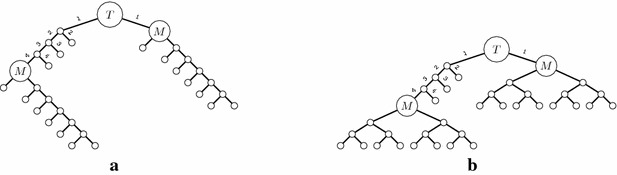
Two synthesis plans for *T* which for both occurrences of *M* use either bond set $M'$ or bond set $M''$. The plan in **a** has ${{\mathrm{EPL}}}= 98$, the plan in **b** has ${{\mathrm{EPL}}}= 97$. Both values are larger than the ${{\mathrm{EPL}}}$ for the plan in Fig. 7b

*External Path Length* Recall from “Synthesis planning basics” section that the quality measure external path length (${{\mathrm{EPL}}}$) [8] is the sum of the lengths of all paths from the root to the leaves. When trying (and failing) to express ${{\mathrm{EPL}}}$ as an additive weight function, we discovered a certain peculiarity inherent in the measure: the optimal synthesis plan for a molecule depends on how the molecule is later used. More precisely, what constitutes the best (sub-)synthesis plan for an intermediate molecule inside a larger synthesis plan depends on *where* in the large plan the molecule is used. This does not correspond to physical reality, since different instances of a molecule are not distinguished after creation. As a consequence, in an optimal plan (w.r.t. ${{\mathrm{EPL}}}$) where the same intermediate molecule appears twice, the plan could ask for different subplans for it. As we argued at the start of the “Results” section, such a plan would never be used in practice.

We now demonstrate the above inconsistency by an example, expressed using unary-binary trees (the model in which ${{\mathrm{EPL}}}$ was originally defined [8]).

Consider a (hypothetical) molecule *M*, and assume it can be synthesized using two different bond sets $M'$ and $M''$, as depicted in the top row of Fig. 6. $M'$ admits only linear synthesis plans, whereas $M''$ has many, including a fully convergent one. These synthesis plans are depicted in the bottom row of Fig. 6.

For synthesizing *M* by itself, the measure ${{\mathrm{EPL}}}$ is minimized by the fully convergent plan for the bond set $M''$. However, consider the molecule *T* depicted in Fig. 7a, with the synthesis plan depicted in Fig. 7b. In this synthesis plan, the molecule *M* appears twice, with different sub-synthesis plans. According to ${{\mathrm{EPL}}}$ these different synthesis plans for *M* are indeed the optimal choices at these two positions in the remaining plan, as the reader can readily verify. For instance, the two alternatives shown in Fig. 8a, b have ${{\mathrm{EPL}}}=98$ and ${{\mathrm{EPL}}}=97$, while that of Fig. 7b has ${{\mathrm{EPL}}}=96$. In other words, according to ${{\mathrm{EPL}}}$, the position of *M* in the large plan determines how it should be made. We note that already in [8], Hendrickson expressed some reservations about the reliability of the measure as a cost function, but in less explicit terms than the phenomenon demonstrated above.

## Discussion

**Fig. 9:**
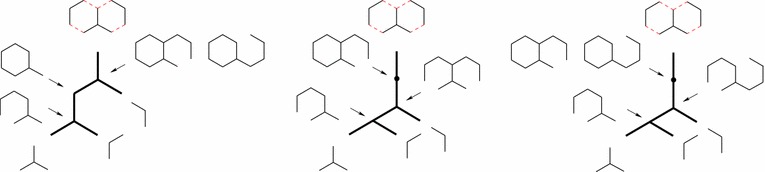
An example bond set of size four for decalin. It leads to five synthesis plans, distributed over two different unary-binary tree topologies. This is the only bond set of size four leading to five synthesis plans

**Fig. 10:**
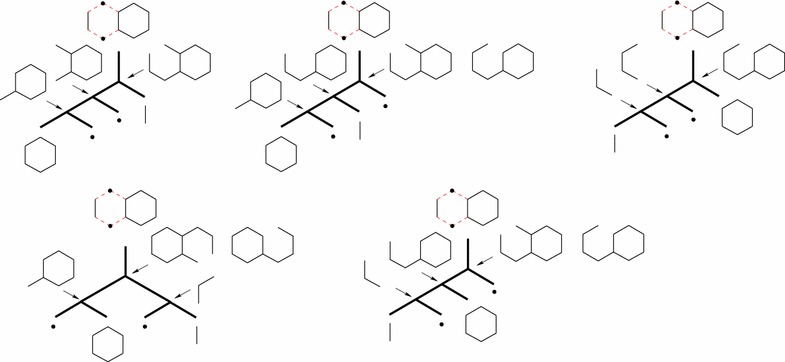
An example bond set of size four for decalin. It leads to eight synthesis plans, distributed over two different unary-binary tree topologies. This is the only bond set of size four leading to eight synthesis plans

In this section, we discuss our approach in a practical context. Using decalin as an example, we first show that even small molecules admit a large number of synthesis plans. We next investigate how sensitive the ordering of these plans is to changes in the yields of reactions. Finally, we compare a formal synthesis plan to a fully detailed synthesis plan from the literature, illustrating the difference in level of details included. All three aspects demonstrate that having *K* good synthesis plans available is a clear advantage over having just the single best plan.

An implementation of the algorithms in “Appendix 2: The *K* best plans algorithm” and “Appendix 3: Bond set based HoR construction” can be acquired upon request by email to the authors.

### Number of synthesis plans for decalin

Recall that decalin is a bicyclic organic molecule with ten carbon atoms (Fig. 1). It has previously been used as an example molecule in graph theoretic approaches to synthesis planning [6]. Below, we use it to demonstrate that even small molecules typically lead to a large variety of synthesis plans.

We consider synthesis plans based on bond sets up to size four. A bond set can be represented as an edge-colored molecule graph, in which red edges are in the bond set and black edges are not. From this, the number of non-isomorphic bond sets of a certain size can be calculated by a straightforward application of Pólya’s Enumeration Theorem [33]: decalin has four non-isomorphic bond sets of size one, 18^2^ non-isomorphic bond sets of size two, 47 non-isomorphic bond sets of size three, and 92 non-isomorphic bond sets of size four.

**Fig. 11:**
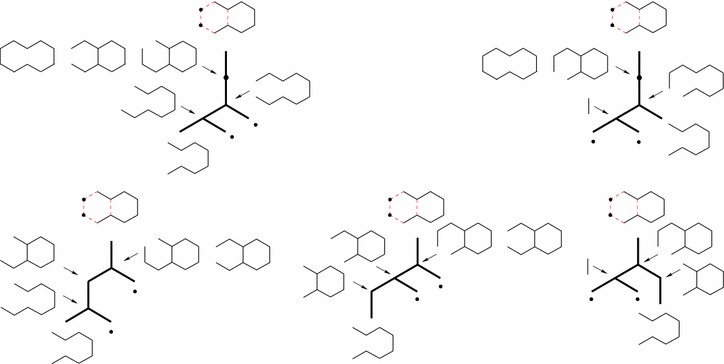
An example bond set of size four for decalin. It leads to ten synthesis plans, distributed over four different unary-binary tree topologies. There are nine other bond sets of size four leading to ten synthesis plans

For each of the 92 bond sets of size four, we count how many different plans this leads to. We do this by creating the ${{\mathrm{HoR}}}$ based on the bond set in question (using the algorithm in “Appendix 3: Bond set based HoR construction”) and then computing the *K* shortest hyperpath for $K = \infty$ (using the algorithm in “Appendix 2: The *K* best plans algorithm”). Of these 92 bond sets, two lead to three different synthesis plans each (these are depicted in Fig. 1), one leads to five different synthesis plans (Fig. 9), one leads to eight synthesis plans (Fig. 10), and the rest each leads to at least ten possible synthesis plans (one example is given in Fig. 11). The maximum number for a single bond set is 38 different plans. The total number of plans in the collection is 1711.^3^

In more detail, consider the example of the bond set depicted in Fig. 10. It gives rise to eight different synthesis plans, distributed over two different unary-binary tree topologies. Considering leaf vertex labels only (as often done in chemical literature) there are five different labeled unary-binary tree topologies. However, for three out of these five leaf-vertex-labeled trees, one internal vertex may have two different labels, which leads to the final eight different synthesis plans.

From numbers above, we see that even small molecules can have a large number of possible synthesis plans. For larger molecules, an exhaustive enumeration becomes very costly in terms of computation time, and having a polynomial time algorithm for returning the *K* best is a strong asset.

### Order of synthesis plans

We now add retro yields to the synthesis plans of decalin. For each bond set, two different sets of example retro yields were used: one for which $\sum _{v\in T(e)} r_{v,e} = 1.25$ for each hyperarc *e* representing a reaction, and one for which $\sum _{v\in T(e)} r_{v,e} = 2.5$. These two cases correspond to a yield of $1/1.25 = 80\%$ and $1/2.5 = 40\%$, respectively. For each reaction, the retro yields are distributed between the reactants in proportion to their number of carbon atoms.

As an example, consider the three synthesis plans of Fig. 1a. With a yield of 80% in each reaction, one can verify that the total weight of starting materials needed to create one gram of decalin is 2.27 g in the case where the first affixation is followed by a cyclization, and 2.34 g in the two cases where both cyclization reactions are performed in the end. With a yield of 40%, the corresponding numbers are 32.5 and 34.4 g. Fig. 5 shows the ${{\mathrm{HoR}}}$ of these three plans decorated with the retro yields corresponding to 80% yield.

As another example, consider the eight plans in Fig. 10. Using a yield of 80% in each reaction, one can verify that the best plan is the top leftmost plan. This has a total weight of starting materials of 1.87 g. With a yield of 40%, the best plan is the top rightmost plan with a total weight of starting materials of 15.63 g.

From all synthesis plans of all possible bond sets of size 4, the best possible total weight turns out to be 1.72 g if the yield is 80%, and 10.0 g if the yield in each reaction is 40% (plans not among those depicted).

We now try to quantify how much the total ordering among all the plans changes when switching between these two sets of retro yields. This will give information on how sensitive the ranking of plans is to changes in the yields of reactions. For a given bond set, the two yield values 80 and 40% used above each gives rise to a ranking of the synthesis plans of the bond set. Let *i* be the first position where these rankings disagree, i.e., the first $i-1$ best plans are the same in the two rankings, but the *i*’th plan is not. For each possible value of *i*, we count how many of the 92 different bond sets have this value as the first position where the rankings disagree. These counts are listed in Table 1 as ${{\mathrm{Count}}}(i)$.

**Table 1:** Illustrating the sensitivity of the plans to changes in the yield values from 80 to $40\%$

*i*	1	2	4	5	10	Same ranking	Total
${{\mathrm{Count}}}(i)$	1	7	2	4	1	77	92

Each entry ${{\mathrm{Count}}}(i)$ is the number of bond sets of size four for which the $i-1$ best plans are the same, but the *i*th plan is not

When planning, yields are often not known with high precision (and may even change over time as lab experience with the chosen reactions grows). The above shows that some plans may be quite sensitive to the exact yield values. Running our algorithm for two different sets of yield values and a fairly large *K*, and then taking the intersection of the results, is a way to learn which among the good plans are robust towards uncertainties in the yield values.

### Detailed chemical synthesis plan for a size 2 bond set for decalin

**Fig. 12:**
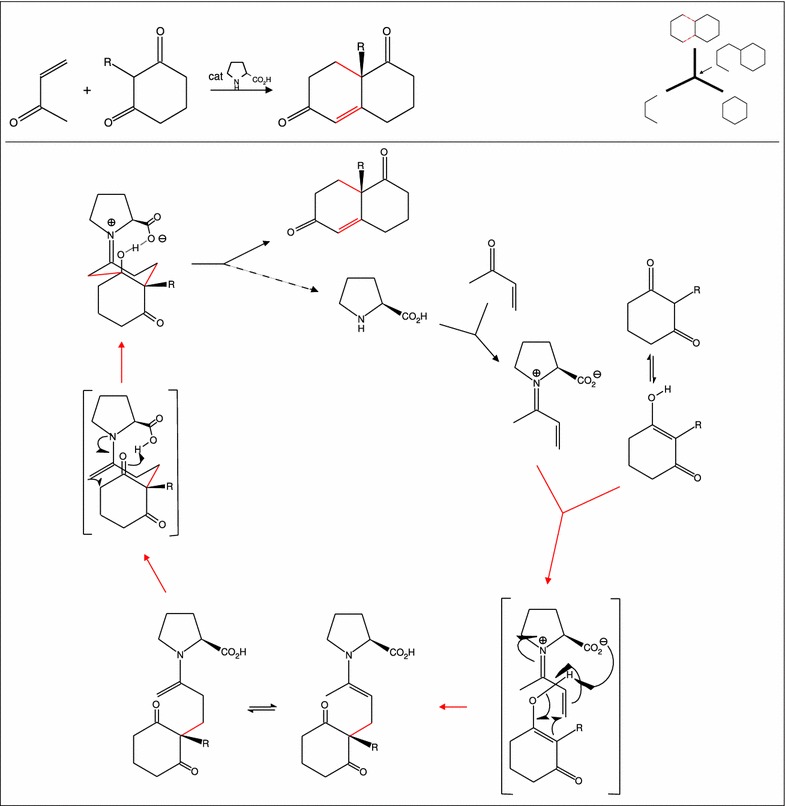
Top left: Overall reaction for the synthesis of Wieland-Miescher Ketone (WMK) from methyl vinyl ketone and 2-methylcyclohexane-1,3-dione. Top right: The phase one synthesis plan corresponding to this overall reaction. It has decalin as skeleton molecule and a bond set of size two. Bottom: Detailed reaction mechanism of the L-proline catalyzed Robinson annulation to yield WMK. The red arrows indicate the affixation and acclimatization steps in the synthesis tree (top right). Note that both bond fixes require a lot of electron rearrangement shown as arrows in the two bracketed reaction transition states. In all subfigures, the bonds of the bond set are shown by red lines

As discussed earlier, synthesis planning often proceeds in two phases, the first of which is identification of a set of construction reactions, and the second is consideration of functionalization reactions.

In this section, we want to illustrate the difference between a skeleton plan from the first phase and a detailed synthesis plan including the functionalization reactions from the second phase. As will be apparent, the difference can be large. Hence, the single best plan from phase one may easily turn out to be infeasible under actual lab conditions. Being able to find the *K* best plans in phase one gives a much more robust strategy, since this gives a number good plans on which practitioners can build in phase two.

We use the synthesis of the Wieland-Miescher Ketone (WMK) as an example. WMK is a key building block [34] in the total synthesis of numerous natural products possessing a wide spectrum of biological activity. The reaction (also known as Hajos-Parrish-Eder-Sauer-Wiechert reaction) is one of the first examples of asymmetric organocatalysis. The overall reaction is depicted in the top left of Fig. 12. It corresponds to the phase one plan shown in top right of Fig. 12, which has decalin as skeleton molecule and a bond set of size two. Interestingly, only the shown size two bond set has been under heavy investigation, and many different organocatalysts have been tried out to improve the yield and enatiomeric access of the reaction. Alternative bond sets, however, have only occasionally been tried out, which is surprising given the central role of WMK as a versatile building block in natural product synthesis. The affixation and cyclization steps require quite heavy valence electron rearrangements as illustrated by arrows in the two bracketed transition states in the lower part of Fig. 12. Furthermore, the synthetic target WMK is garnished with functional groups and chiral centers which are not considered in the phase one plan.

## Conclusions

We have demonstrated that a core part of chemical synthesis planning can be phrased as a combinatorial optimization problem on hypergraphs by modeling individual synthesis plans as directed hyperpaths embedded in a hypergraph of reactions (${{\mathrm{HoR}}}$) representing the chemistry of interest. An immediate consequence is that the *K* best synthesis plans for a given target can be computed in polynomial time [20] for a number of quality measures of practical importance, and indeed for any measure which can be expressed as an additive weight function. The polynomial runtime makes it feasible to do this even for big *K* and large molecules. Having *K* good plans to choose from has many benefits: it makes the synthesis planning process much more robust towards actual feasibility when in later stages adding functionalization reactions and other chemical details, it allows one to combine several quality measures, and it provides a way to deal with imprecise yield estimates.

Looking at the standard retrosynthetic approach, the obvious type of ${{\mathrm{HoR}}}$ is defined using a fixed target and a bond set, cf. the algorithm described in “Appendix 3: Bond set based HoR construction”. However, our modeling is not restricted to this. For instance, it is possible to combine any number of known synthesis plans for a target molecule into a ${{\mathrm{HoR}}}$, from which new hybrid plans may arise. More generally, any database of reactions and starting materials can be used to define a ${{\mathrm{HoR}}}$.

Along the way, we also demonstrated that the classic quality measure ${{\mathrm{EPL}}}$ has a built-in inconsistency which could render its use in synthesis planning questionable.

The work presented here is one step towards improving chemical synthesis planning in the light of developments in graph and hypergraph algorithms. A natural next step would be to attempt to add more chemical detail in the modeling. For instance, one could try to include refunctionalization reactions and to consider strategies for introducing and removing protective groups within this modeling framework.

## Figures and Tables

**Fig. 13:**
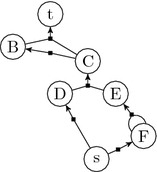
Hyperpath

**Fig. 14:**
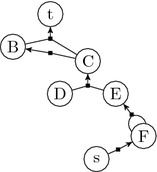
Not hyperpath

**Fig. 15:**
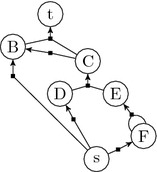
Not hyperpath

**Fig. 16:**
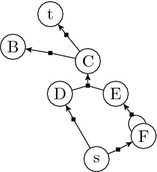
Not hyperpath

**Fig. 17:**
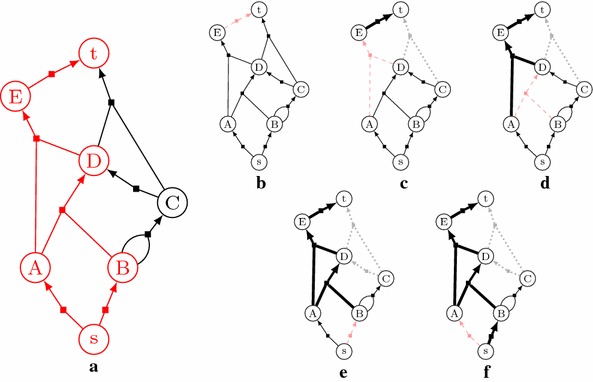
Figure illustrating algorithm Back-Branch. Within the graph *H* a hyperpath is marked red. Branching is performed along this hyperpath using the hyperarc order $\langle p(A),p(B),p(D),p(E),p(t) \rangle$. The resulting hypergraphs $H^5, H^4, \ldots , H^1$ are also depicted. Thick hyperarcs are fixed, which results in the deletion of the gray, dotted hyperarcs. The red, dashed hyperarc in each hypergraph is the hyperarc at which the deviation takes place, and thus, this hyperarc is also deleted. **a** *H*. **b** $H^5$. **c** $H^4$. **d** $H^3$. **e** $H^2$. **f** $H^1$

**Fig. 18:**
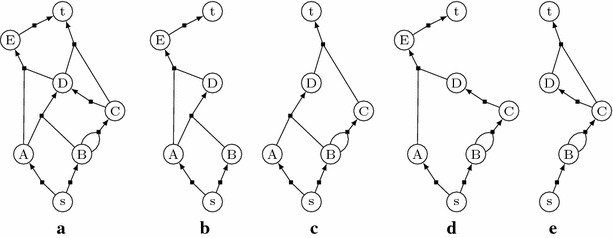
A graph *H* along with its 4 hyperpaths $\pi _1, \pi _2, \pi _3, \pi _4$. **a** *H*. **b** $\pi _1$. **c** $\pi _2$. **d** $\pi _3$. **e** $\pi _4$

**Fig. 19:**
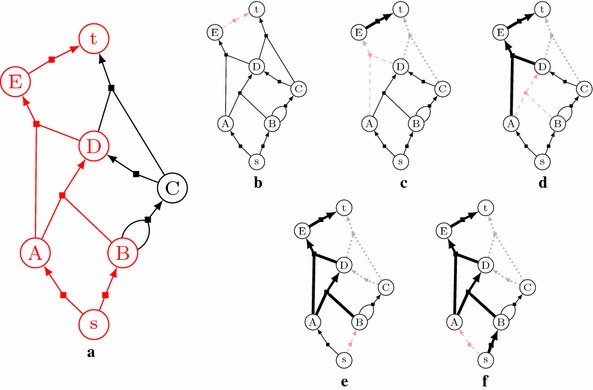
Branching on *H* along the hyperpath $\pi _1$. In **a** $\pi _1$ is highlighted in *H* with the color red. The rest of the figure illustrates the backwards branching. Each subfigure **b**–**f** shows a graph $H^i$ and how it is created from *H*. Dashed and dotted hyperarcs are *not* part of the graphs, but were deleted due to branching. For each $H^i$, the deleted hyperarc $p(v_i)$ is red and dashed. Each fixed hyperarc is thick, and if fixing any hyperarc has led to deletion of other hyperarcs, these are gray and dotted. Furthermore, the caption for each subfigure indicates the set of hyperpaths represented by that graph. **a** *H*: {*π*_1_, *π*_2_, *π*_3_, *π*_4_}, *π*_1_ is red. **b** *H*^5^: {*π*_2_, *π*_4_}, **c** *H*^4^: $\emptyset$. **d** *H*^3^: {*π*_3_}. **e** *H*^2^: $\emptyset$. **f** *H*^1^: $\emptyset$

**Fig. 20:**
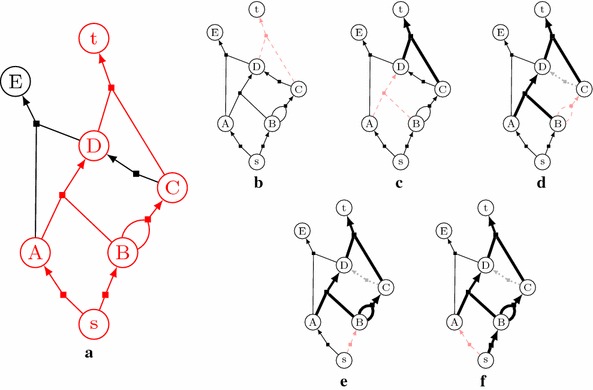
Branching on $H^5$ along the hyperpath $\pi _2$. In **a** $\pi _2$ is highlighted in $H^5$ with the color red. The rest of the figure illustrates the backwards branching. Each subfigure **b**–**f** shows a graph $H^{5i}$ and how it is created from $H^5$. Dashed and dotted hyperarcs are *not* part of the graphs, but were deleted due to branching. For each $H^{5i}$ the deleted hyperarc $p(v_i)$ is red and dashed. Each fixed hyperarc is thick, and if fixing any hyperarc has led to deletion of other hyperarcs, these are gray and dotted. Furthermore, the caption of each subfigure indicates the set of hyperpaths represented by that graph. **a** *H*^5^: {*π*_2_, *π*_4_}, *π*_2_ is red. **b** *H*^55^: $\emptyset$, **c** *H*^54^: {*π*_4_}. **d** *H*^53^: $\emptyset$, **e** *H*^52^: $\emptyset$. **f** *H*^51^: $\emptyset$

## Appendix 1: Hyperpaths

Examples of hyperpaths and non-hyperpaths. Figure 13 is a hyperpath from *s* to *t*: each vertex (except *s*) has exactly one ingoing hyperarc, each vertex (except *t*) has at least one outgoing hyperarc, and the ordering $\langle p(F), p(E), p(D), p(C), p(B), p(t)\rangle$ of the hyperarcs meets the condition of Definition 2(1). Figures 14, 15, and 16 are not hyperpaths: in Fig. 14, the vertex *D* has no ingoing hyperarc, in Fig. 15 the vertex *B* has two ingoing hyperarcs (a hyperpath can be obtained by deleting either of these hyperarcs), and in Fig. 16 the vertex *B* has no outgoing hyperarcs (a hyperpath can be obtained by deleting this vertex together with the incident hyperarc).

## Appendix 2: The ***K*** best plans algorithm

As mentioned, a polynomial time algorithm for finding the *K* best hyperpaths in a hypergraph was given by Nielsen et al. [20]. We here explain the algorithm and illustrate how it works by an example.

Let $H=(V,E)$ be a directed B-hypergraph, let $s,t\in V$ be vertices for which there exists at least one hyperpath from *s* to *t*, let $\mathcal {P}$ be the set of all hyperpaths from *s* to *t*, and let *W* be an additive weight function on hyperpaths, cf. Eq. (2). We assume the existence of an algorithm ShortestPath for computing a (single) optimal hyperpath according to *W*. Let $\pi _{st}$ be such an optimal hyperpath from *s* to *t* in *H*.

In the algorithm of Nielsen et al., the ordering $\langle p(v_1), p(v_2),\dots , p(v_{q-1}), p(t)\rangle$ of the hyperarcs of $\pi _{st}$ is used^4^ to partition the remaining hyperpaths into *q* disjoint subsets $\mathcal {P}^i$, $1\le i\le q$, as follows (where $v_q$ denotes *t*): $\mathcal {P}^i$ is the set of all hyperpaths from *s* to *t* containing the hyperarcs $p(v_{i+1}),p(v_{i+2}),\dots ,p(v_{q-1}),p(t)$ and not containing $p(v_{i})$. In other words, each $\mathcal {P}^i$ consists of all *st*-hyperpaths that have the same last $q-i$ hyperarcs as $\pi _{st}$ and deviate from $\pi _{st}$ exactly at the *i*th hyperarc. Clearly, the $\mathcal {P}^i$s form a partitioning of $\mathcal {P}{\setminus } \{\pi _{st}\}$.

The idea behind the algorithm is to view this partition of $\mathcal {P}{\setminus }\{ \pi _{st}\}$ along $\pi _{st}$ as a set of *q* new optimal hyperpath problems. Each set $\mathcal {P}^i$ has an optimal hyperpath $\pi ^i$, and the best of these, say $\pi _j$, will be the optimal of the remaining hyperpaths $\mathcal {P}{\setminus } \{\pi _{st}\}$, i.e., the second best hyperpath in *H*. Now, output $\pi _j$ and partition $\mathcal {P}^j{\setminus } \{\pi _j\}$ along $\pi _j$. To find the third best hyperpath, note that the sets of this partition together with the remaining sets $\mathcal {P}^i$ for $i=1,2 \dots j-1,j+1 \dots q$ form a partition of $\mathcal {P}{\setminus } \{ \pi _{st}, \pi _j\}$. Hence, the process can be continued in the same fashion, outputting the hyperpaths in *H* in sorted order.

Storing $\mathcal {P}$ or $\mathcal {P}^i$ as actual sets of hyperpaths requires precomputation of all hyperpaths, which is undesirable. Instead, each set of *st*-hyperpaths can be represented by a subhypergraph of *H* [20]. *H* itself naturally contains all *st*-hyperpaths of *H* and is thus the hypergraph representation of the set $\mathcal {P}$. Each $\mathcal {P}^i$ is represented by a hypergraph $H^i=(V^i,E^i)$ obtained by *H* in the following manner:

- The vertex set remains the same, i.e., $V^i=V$.
- The arc set $E^i$ is obtained from *E* as follows:
  - The hyperarc $p(v_i)$ is removed.
  - The hyperarcs $p(v_{i+1}),p(v_{i+2}),\ldots ,p(v_{q-1}),p(t)$ are *fixed* in $H^i$. By this we mean that for each $v_j, j > i$, all ingoing hyperarcs of $v_j$ except $p(v_j)$ are removed, making $p(v_j)$ the only ingoing hyperarc to $v_j$.

It is shown in [20] that $\pi ^i \in \mathcal {P}^i$ if and only if $\pi ^i$ is a *st*-hyperpath in $H^i$.

The partition of a set of hyperpaths $\widetilde{\mathcal {P}}$ along a hyperpath $\widetilde{\pi } \in \widetilde{\mathcal {P}}$ is computed by the backwards branching procedure Back-Branch, Algorithm 2. It takes as input the hypergraph representation $\widetilde{H}$ of $\widetilde{\mathcal {P}}$ and the hyperpath $\widetilde{\pi } \in \widetilde{H}$ with *q* hyperarcs and predecessor function *p*. Each $\widetilde{H}^i$ is computed by deleting $p(v_i)$ and fixing the subsequent hyperarcs as described above (in Algorithm 2, ${{\mathrm{BS}}}(v)$ denotes the set of ingoing hyperarcs for vertex *v*). Finally, the set $\mathcal {B}=\{\widetilde{H}^i \mid 1\le i \le q\}$ is returned.

This algorithm is illustrated in Fig. 17. Within the graph *H* a hyperpath is marked red. Branching is performed along this hyperpath using the hyperarc order $\langle p(A),p(B),p(D),p(E),p(t) \rangle$. The resulting hypergraphs $H^5, H^4, \ldots , H^1$ are also depicted. Thick hyperarcs are fixed, which results in the deletion of the gray, dotted hyperarcs. The red and dashed hyperarc in each graph is the hyperarc at which the deviation takes place, and thus, this hyperarc is also deleted.

The main algorithm K-Shortest, Algorithm 3, maintains a priority queue *L* of tuples of the form $(\widetilde{H}, \widetilde{\pi })$, where $\widetilde{H}$ is a hypergraph representation of a set of *st*-hyperpaths in *H*, as described above, and $\widetilde{\pi }$ is the best of these according to *W*. In the priority queue, the key of a tuple is $W(\widetilde{\pi })$. Initially *L* contains the tuple $(H,\pi _{st})$.

In each iteration, the smallest tuple of *L*, say $(H', \pi ')$, is extracted and $\pi '$ is output as the next best hyperpath of *H*. Then $H'$ is partitioned along $\pi '$ using the backwards branching procedure Back-Branch, and each new partition along with its optimal hyperpath is inserted into *L* (unless no hyperpath exists in the partition). The algorithm terminates when *K* hyperpaths have been output or if *L* is empty.

The correctness of the algorithm K-Shortest follows from the fact that it maintains the following invariant: At the end of iteration *i*, the *i* best hyperpaths $\pi _1, \pi _2, \ldots , \pi _i$ have been output, and the hypergraphs of the tuples of *L* represent a partition of $\mathcal {P}{\setminus } \{ \pi _1, \pi _2, \ldots , \pi _i\}$.

Algorithm ShortestPath in line 12 of Algorithm 3 computes the optimal hyperpath according to *W*. In acyclic B-hypergraphs this can be done using dynamic programming in $O(|V|+{{\mathrm{size}}}(H))$ time [35].

For acyclic hypergraphs, algorithm K-Shortest runs in $O(K|V|(|V|+{{\mathrm{size}}}(H)))$ time [20]. As described earlier, for a ${{\mathrm{HoR}}}$ *H* we have ${{\mathrm{size}}}(H)\le 3|E|$, which means that when using the algorithm for synthesis planning the runtime is $O(K|V|(|V|+|E|))$.

### *Example*

The hypergraph *H* of Fig. 17 is depicted again in Fig. 18a. There are four hyperpaths in this hyperpath, namely $\pi _1, \pi _2, \pi _3$ and $\pi _4$, depicted in Fig. 18b, e. We illustrate how the best three hyperpaths are computed by algorithm K-Shortest when assuming $W(\pi _1)< W(\pi _2)< W(\pi _3)<W(\pi _4)$.

Initially, $L=\{(H,\pi _1)\}$. The tuple is extracted, $\pi _1$ is output as the optimal hyperpath of *H*, and branching is performed on *H* along $\pi _1$. The branching is shown in Fig. 19 ($\pi _1$ is red). This figure is identical to Fig. 17 except for added information on the set of hyperpaths in each hypergraph. For instance, $H^5$ contains the hyperpaths $\pi _2$ and $\pi _4$. The optimal hyperpath is computed for each $H^i$, and since $H^1,H^2$ and $H^4$ have no hyperpaths from *s* to *t*, $L=\{(H^5,\pi _2),(H^3,\pi _3)\}$ by the beginning of iteration two.

In iteration two, the tuple $(H^5,\pi _2)$ is extracted since we assumed that $W(\pi _2)<W(\pi _ 3)$. The hyperpath $\pi _2$ is output as the second best hyperpath in *H* and branching is performed on $H^5$ along $\pi _2$. This is shown in Fig. 20 ($\pi _2$ is red). The only hypergraph in which there is a hyperpath from *s* to *t* is $H^{54}$. Hence, the tuple $(H^{54},\pi _4)$ is inserted into *L* which becomes $L=\{ (H^3,\pi _3), (H^{54},\pi _4)\}$.

In iteration three, the tuple $(H^3,\pi _3)$ is extracted from *L*, $\pi _3$ is output as the third best hyperpath of *H* and the algorithm terminates.

## Appendix 3: Bond set based HoR construction

In this appendix, we consider the case of a HoR for which the sets *R* and *S* of Definition 3 are defined by recursively breaking bonds in a given subset *B* of the bonds of the target in all possible ways, and present algorithmic details of how to do this efficiently.

A straight-forward method would be to consider all the |*B*|! orders of fixing the bonds in *B*, each of which gives a unary-binary tree, and then for each tree enforcing the requirement that no intermediate molecule is synthesized in more than one way, cf. Fig. 2. Enforcing that requirement (and creating the DAG of Fig. 3b) can be done as follows: for each intermediate molecule appearing more than once in vertices of the tree choose one of these vertices and change all pointers to the rest of these vertices to point to that chosen vertex. To create all possible synthesis plans, for each such intermediate molecule all choices should be considered. This should be done in a bottom-up fashion, from smaller intermediate molecules to larger ones, in an ordering where molecule $m_1$ is considered larger than molecule $m_2$ if when seen as graphs $m_1$ has more vertices than $m_2$, or they have the same number of vertices and $m_1$ has more edges than $m_2$. Finally, *R* and *S* are set to the union of the reactions, respectively starting materials, of all of the synthesis plans created.

This process, however, would take $\Omega (|B|!)$ time. It would also render pointless the use of the algorithm for finding the *K* optimal hyperpaths in the resulting hypergraph, since having considered each synthesis plan explicitly one could just as well have evaluated the cost of each in the process, while keeping track of the *K* best.

We now give an algorithm Expand${{\mathrm{HoR}}}$ (see Algorithm 4) for constructing the ${{\mathrm{HoR}}}$ which under reasonable assumptions runs in $O(|V| \cdot |B| \cdot |t|)$ time, where |*t*| is the size (number of vertices plus number of edges when seen as a graph) of the target molecule *t*. The algorithm explores the possible synthesis plans in a top-down manner, starting with the target molecule, and recursively breaking bonds from *B*. To avoid exploring the synthesis of a given intermediate molecule more than once, it checks if an intermediate molecule has already been explored before recursing on it.

Each intermediate molecule *m* is represented as a labeled (standard) graph, with the edges inside *m* corresponding to bonds in *B* being marked. Such a graph can be traversed in *O*(|*m*|) time, and for each bond in *B*, the intermediate molecules produced by removing this bond can be found in *O*(|*m*|) time, as they are the connected components of the resulting graph.

A molecule can also be represented by a unique string identifier [36, 37] for molecules/labeled graphs. In the algorithm we use two types of unique string identifiers for intermediate molecules: IDbond(*m*) for the graph of the molecule *m* including the marking on edges of the bonds from *B*, and ID(*m*) where these markings are disregarded. The reason is that it is possible for a bond set *B* to specify, in different locations of the target molecule, the same intermediate molecule with different internal sets of bond set edges. In any synthesis plan, if this intermediate molecule appears, it should be produced in only one way.^5^ However, for considering all possible synthesis plans, all the ways of synthesizing this intermediate molecule should be considered. In other words, a intermediate molecule appearing in several places should be represented by a single vertex *v* in the ${{\mathrm{HoR}}}$, but its synthesis should be explored for all the occurring subsets of bond set edges inside it. Therefore, the check in the algorithm for further exploration of an intermediate molecule *m* is based on whether IDbond(*m*) has been seen before, but the vertices of the resulting ${{\mathrm{HoR}}}$ hypergraph are based on the ID(*m*) values.

A hyperarc $e = (v, v_1, v_2)$ is a triple of string identifiers of type ID(*m*), with *v* representing the output molecule, and $v_1$ and $v_2$ representing the input molecules of the reaction modeled by *e*. When $|T(e)| = 1$, $v_2$ is the empty string. The identifiers of type IDbond(*m*) seen so far, as well as the generated edges, are kept in hash tables $V'$ and *E*, respectively. At the end of the algorithm, *V* can be generated as all vertices appearing in hyperarcs in *E*. The ${{\mathrm{HoR}}}$ is then the hypergraph (*V*, *E*).

The algorithm Expand${{\mathrm{HoR}}}$ maintains the invariant that at the time of call Expand${{\mathrm{HoR}}}$(*m*), IDbond(*m*) has not been explored before. The very first call has *m* equal to the target molecule. The sets $V'$ and *E* are global variables, each initialized to the empty set. When inserting into a hash table, it is assumed that nothing happens if the value is already present. The value *S* is just an identifier for the dummy vertex *s* of the ${{\mathrm{HoR}}}$.

Each vertex *v* in the resulting hypergraph may represent an intermediate molecule on which the algorithm is called several times, with different values of IDbond(*m*), but same value of ID(*m*). For each value of IDbond(*m*), there can only be one call, due to the maintenance of $V'$. If we assume that these values represents occurrences of the intermediate molecule at non-overlapping places in the target molecule, their combined number of bond set edges is bounded by |*B*|. Under this assumption, each recursive call *from* an vertex *v* (with id ID(*m*)) is induced by a different bond set edge in *t*, so we can only have |*B*| recursive calls in total from *v*, hence only $O(|V| \cdot |B|)$ recursive calls in total in the algorithm. For each bond edge, the connected components resulting from its removal can be found in *O*(|*t*|) time. Hence, the algorithm uses $O(|V| \cdot |B| \cdot |t|)$ time, if the time for finding string identifiers is assumed to be also *O*(|*t*|). The time for finding a unique string identifier is in the worst case actually exponential in the size of the intermediate molecule (consistent with the fact that finding unique identifiers solves the graph-isomorphism problem, which is not known to be solvable in polynomial time), but empirically, real-life molecules do not represent worst case instances, since algorithms for this work very fast in practice [37]. Note that a similar assumption on the time for solving the graph-isomorphism on real-life molecules applies to the straight-forward algorithm described above, since it also needs to check whether intermediate molecules are the same. Hashing operations takes *O*(1) time, but only in the expected sense, hence the bound for the algorithm stated above is expected time.

We note that |*V*| is always at most $O(2^{|B|})$, since each $v \in V$ can be specified by the bonds in *B* which are fixed in the subtree of *v* in some synthesis plan. Despite being exponential in |*B*|, this is much better than $\Omega (|B|!)$. For certain target molecules, |*V*| can be bound even better—for instance, for linear molecules |*V*| is $O(|B|^2)$.

## Appendix 4: Total weight of starting materials

We here show the connection between the two variants of the quality measure total weight of starting materials (${{\mathrm{TW}}}$) given by Hendrickson [8] and by Smith [9].

Recall the definition from “Synthesis planning basics” section of Smith’s version (here slightly rephrased in order to prepare for the inductive proof of Proposition 1 below): Let $T_t$ be the unary-binary tree representation of a synthesis plan for a target molecule *t*, let $P_{ti}$ be the path in $T_t$ from *t* to leaf *i*, and for an edge $e=(u,v)$ in $T_t$ let $r_e$ be the weight in grams of molecule *v* needed to create one gram of *u*. Then Smith [9] calculates the total weight of starting materials needed to create one gram of target *t* as follows.^6^

$$\begin{aligned} S_t = \sum _{i \,\text {in}\, T_t} \prod _{e\in P_{ti}} r_e \end{aligned}$$

Hendrickson [8] calculates the total weight of starting materials needed to produce one molecule of the target as follows, where *i* again denotes a leaf in $T_t$.

$$\begin{aligned} H_t = \sum _{P_{ti}\in T_t} w_ix^{|P_{ti}|} \end{aligned}$$

In this formula, Hendrickson assumes the same yield (in the usual chemical meaning, percentage of input material that becomes output material) *y* for all reactions and sets $x=1/y$. The weight of the molecule in vertex *v* is denoted $w_v$. Clearly, for a binary vertex *v* with children $v_1,v_2$ we have $w_v=w_{v_1}+w_{v_2}$ and for a unary vertex *v* with child $v_1$ we have $w_{v}=w_{v_1}$.

The following proposition shows that given *x* in Hendrickson’s definition, we can set the retro yields in Smith’s definition such that the two measures have proportional values. Hence, Smith’s definition is a generalization of Hendrickson’s.

### **Proposition 1**

*Let *$r_{(u,v)} = \frac{w_v}{w_u} x$* for all edges *(*u*, *v*)* in the unary-binary tree* $T_t$* representing a synthesis plan for* *t*.* Then* $S_t w_{t} = H_t$.

### *Proof*

We prove this by induction over the height of the unary-binary tree.

Consider the base case where the height $h=0$: the unary-binary tree consists of just a target *t*. Then $S_w=1$ and $H_w=w_t$ so $S_w w_t = H_w$.

For the inductive step, let $h > 0$ and assume $S_{t'} w_{t'} = H_{t'}$ for any target $t'$ with a unary-binary tree of height $k<h$. There are two cases: (i.) *t* is the product of a cyclization and has one child, or (ii.) *t* is the product of an affixation and has two children.

Case (ii): Let the children of *t* be *v*, *u*. We have

$$\begin{aligned} r_{(t,v)} = \frac{w_v}{w_t}x =\frac{w_v}{w_v+w_u}x\\ r_{(t,u)} = \frac{w_u}{w_t}x =\frac{w_u}{w_v+w_u}x \end{aligned}$$

From $S_t= r_{(t,v)}S_{v}+r_{(t,u)}S_{u}$ and $H_t= x(H_{v}+ H_u)$ we get

$$\begin{aligned} S_t w_t&= (r_{(t,v)}S_{v}+r_{(t,u)}S_{u}) w_{t} \\&= \left( \frac{w_v}{w_v+w_u}xS_{v}+\frac{w_u}{w_v+w_u}xS_{u}\right) w_{t} \\&= x\left( \frac{w_vS_{v} + w_uS_{u}}{w_v+w_u}\right) (w_{v}+w_u), \\&= x\left( w_vS_{v} + w_uS_{u}\right) \\&= x (H_{v} + H_u) \text { [by the inductive hypothesis]} \\&= H_t \\ \end{aligned}$$

Case (i) is similar, only simpler. $\square$

## Footnotes

1 Not to be confused with the simpler concept of (non-hyper) paths in the often used bipartite graph representation of hypergraphs.

2 Note that Bertz [6] also counts bond sets of size two, but does not consider isomorphism issues and hence arrives at a larger number.

3 In this sum, we did not check for isomorphic plans arising from different bond sets.

## References

[1] Corey EJ, Wipke WT (1969). Computer-assisted design of complex organic syntheses. Science.

[2] Hendrickson JB, Braun-Keller E, Toczko GA (1981). A logic for synthesis design. Tetrahedron.

[3] Todd MH (2005). Computer-aided organic synthesis. Chem Soc Rev.

[4] Andraos J (2012). The algebra of organic synthesis: green metrics, design strategy, route selection, and optimization.

[5] Rücker C, Rücker G, Bertz SH (2004). Organic synthesis-art or science?. J Chem Inf Comput Sci.

[6] Bertz SH, Sommer TJ, Hudlicky T (1993). Application of graph theory to synthesis planning: complexity, reflexivity and vulnerability. Organic synthesis: theory and applications.

[7] Hoffmann RW (2009). Elements of synthesis planning.

[8] Hendrickson JB (1977). Systematic synthesis design. 6. Yield analysis and convergency. J Am Chem Soc.

[9] Smith WD (1997) Computational complexity of synthetic chemistry–basic facts. Technical report. http://citeseerx.ist.psu.edu/viewdoc/summary?doi=10.1.1.49.9276. Accessed on Jan 2018

[10] Bertz SH (2003). Complexity of synthetic routes: linear, convergent and reflexive syntheses 1. New J Chem.

[11] Gelernter HL, Sanders AF, Larsen DL, Agarwal KK, Boivie RH, Spritzer GA, Searleman JE (1977). Empirical explorations of SYNCHEM. Science.

[12] Corey EJ, Howe WJ, Orf HW, Pensak DA, Petersson G (1975). General methods of synthetic analysis. Strategic bond disconnections for bridged polycyclic structures. J Am Chem Soc.

[13] Corey EJ, Cheng X (1995). The logic of chemical synthesis.

[14] Nowak G, Fic G, Emmert-Streib F, Dehmer M, Varmuza K, Bonchev D (2012). Generation of chemical transformations: reaction pathways prediction and synthesis design. Statistical modelling of molecular descriptors in QSAR/QSPR.

[15] Hendrickson JB (2002) Generating benign alternative syntheses: the SynGen program, pp 127–144. 10.1021/bk-2002-0823.ch010

[16] Wipke WT, Rogers D (1984). Artificial intelligence in organic synthesis. sst: starting material selection strategies. an application of superstructure search. J Chem Inf Comput Sci.

[17] Hanessian S, Franco J, Larouche B (1990). The psychobiological basis of heuristic synthesis planning man, machine and the chiron approach. Pure Appl Chem.

[18] Mehta G, Barone R, Chanon M (1998). Computer-aided organic synthesis-sesam: a simple program to unravel “hidden” restructured starting materials skeleta in complex targets. Eur J Organ Chem.

[19] Gillet VJ, Myatt G, Zsoldos Z, Johnson AP (1995). Sprout, hippo and caesa: tools for de novo structure generation and estimation of synthetic accessibility. Perspect Drug Discov Des.

[20] Nielsen LR, Andersen KA, Pretolani D (2005). Finding the $$K$$ shortest hyperpaths. Comput Oper Res.

[21] Velluz L, Valls J, Mathieu J (1967). Spatial arrangement and preparative organic synthesis. Angew Chem Int Ed Engl.

[22] Kim SM, Peña MI, Moll M, Bennett GN, Kavraki LE (2017). A review of parameters and heuristics for guiding metabolic pathfinding. J Chem Inform.

[23] Carbonell P, Fichera D, Pandit SB, Faulon J-L (2012). Enumerating metabolic pathways for the production of heterologous target chemicals in chassis organisms. BMC Syst Biol.

[24] Ausiello G, Franciosa PG, Frigioni D (2001). Directed hypergraphs: problems, algorithmic results, and a novel decremental approach. Theoretical Computer Science.

[25] Thakur M, Tripathi R (2009). Linear connectivity problems in directed hypergraphs. Theor Comput Sci.

[26] Yen JY (1971). Finding the $$k$$ shortest loopless paths in a network. Manag Sci.

[27] Gallo G, Longo G, Pallottino S, Nguyen S (1993). Directed hypergraphs and applications. Discr Appl Math.

[28] https://new.reaxys.com. Accessed on Jan 2018

[29] http://www.cas.org/products/scifinder. Accessed Jan 2018

[30] Liu B, Ramsundar B, Kawthekar P, Shi J, Gomes J, Nguyen QL, Ho S, Sloane J, Wender P, Pande V (2017). Retrosynthetic reaction prediction using neural sequence-to-sequence models. ACS Cent Sci.

[31] Segler MHS, Waller MP (2017). Neural-symbolic machine learning for retrosynthesis and reaction prediction. Chem Eur J.

[32] Coley CW, Rogers L, Green WH, Jensen KF (2017). Computer-assisted retrosynthesis based on molecular similarity. ACS Cent Sci.

[33] Pólya G (1937). Kombinatorische anzahlbestimmungen für gruppen, graphen und chemische verbindungen. Acta Math.

[34] Bradshaw B, Bonjoch J (2012). The Wieland–Miescher Ketone: a journey from organocatalysis to natural product synthesis. SYNLETT.

[35] Gallo G, Pallottino S (1992) Hypergraph models and algorithms for the assembly problem. Technical report, Dipartimento di Informatica, Universitá di Pisa, TR-6/92

[36] Heller SR, McNaught A, Stein S, Tchekhovskoi D, Pletnev IV (2013). InChI—the worldwide chemical structure identifier standard. J Chem Inf.

[37] McKay BD, Piperno A (2014). Practical graph isomorphism II. J Symb Comput.

